# Human health risk assessment of heavy metal concentration in surface water of Sosian river, Eldoret town, Uasin-Gishu County Kenya

**DOI:** 10.1016/j.mex.2023.102298

**Published:** 2023-08-01

**Authors:** Emily N. Munene, Nadir O. Hashim, Willis N. Ambusso

**Affiliations:** Physics Department, Kenyatta University, P.O. Box 43844, Nairobi, Kenya

**Keywords:** Water quality and Health risk assessment, Cancer, Non-cancer risks, Chronic daily intake, Hazard quotient

## Abstract

Heavy metal pollution in surface waters has become a major worldwide issue as people tend to settle where there is readily available source of water like a river. This research evaluates the causes, concentration and associated health risks of heavy metals in River Sosiani as it passes through the town of Eldoret. Seven water samples were collected and analysed for zinc (Zn), copper (Cu) and lead (Pb. The results disclosed that Pb concentrations were estimated to be in the range of 0.06 mg/l to 0.23 mg/l, higher than the permitted limit by WHO of 0.01 mg/l. Cu and Zn concentration levels were below the permissible limits. The chronic daily intake (CDI) indicated that total hazard quotient of non-cancer risk of Pb was above one and the total HI values for children were greatly elevated compared to those of adults in the studied area. This showed a high risk in exposure to Pb. Health human risk was assessed and the incremental life cancer risk (ILCR) values of Pb for children and adults in all sites were found to be negligible with values below 10^−6^. However, there is higher cancer and non-cancer risk for children than adults as far as lead metal is concerned. Therefore, measures should be taken in accordance with the standards to prevent potential risk of the river pollution.

• Human activities make a significant contribution to heavy metal pollution to surface waters which is a threat to humans.
• Water from Sosiani River is not safe for use domestically as far as lead metal levels are concerned.
• The results of this study can be used by decision makers to develop measures which can improve the quality of water in the river catchment.

## Graphical abstract

**Figure:**
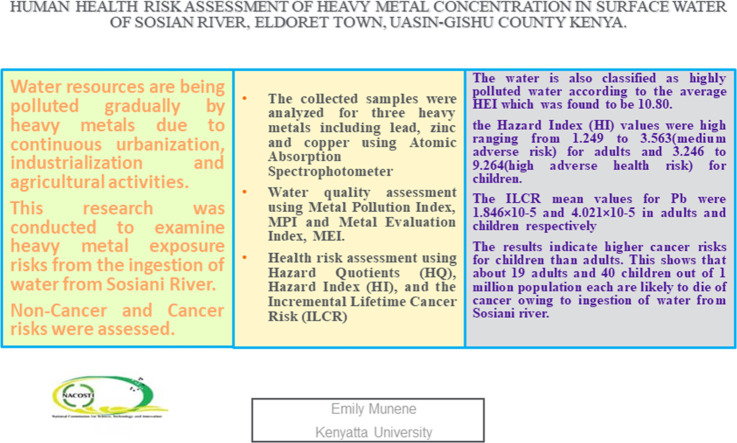

Specifications table

**Table:** 

Subject area:	Environmental Science
More specific subject area:	Health risks of heavy metals in water
Name of your method:	Water quality and Health risk assessment
Name and reference of original method:	Numerical Modeling of Heavy Metals in Riverine Systems in Eldoret, Uasin-Gish County, Kenya. Journal of Agriculture Science & Technology ISSN 1561-7645 JAGST 20 (2) 2021, 1–7.
Resource availability:	The data is available with this article

## Introduction

Water is an essential natural resource necessity for life sustenance. Continuous contamination of fresh water bodies by trace elements has led to less use of water resources for domestic use. Water pollution has become a global environmental problem, especially due to rise in toxicity of heavy metals even at low concentrations [1]. Extended exposure of drinking water to heavy metals has brought about negative long-term effects. Surface water pollution by trace elements is one of the greatest quality issues because of their toxicity nature, increased release and negative impact on human beings. They are increasingly added into the water bodies through human activities like urban runoff, agricultural and industrial effluents, sewage discharge, mining and natural phenomena such as the seepage of underground minerals and soil erosion [2].

Heavy metals like iron, cobalt, copper, zinc manganese and molybdenum are essential in the human body but are toxic at high concentrations [3]. Other metals like lead and mercury and plutonium are toxic even in low concentrations.

Exposure to excessive levels of copper can result to liver and kidney damage, anemia, immunotoxicity, and developmental toxicity [4]. Ingesting larger amounts of zinc can cause anorexia, vomiting, and diarrhea. Chronic toxicity of zinc may result in copper deficiency and may cause nerve damage. Lead toxicity affects the normal functioning of the nervous system and longer exposure causes severe effects on kidney as well as brain [5]. Children absorb higher amounts of lead than adults which is highly dangerous as they are developing [6]. Lead affects the reproductive systems of both males and females [7], where there is a reduction in sperm count and volume in males, Wu et al. [8]. In females, high lead exposure and cause miscarriage, premature birth, low birth weight, and developmental and spontaneous abortion of the fetus [9]. Lead toxicity symptoms can worsen and give rise to paralysis, coma, or even death [10].

Eldoret town is a vibrant town that has experienced a very rapid population increase from about 289,380 to 475, 716 between the years 2009 and 2019 [11,12]. This high rate of urbanization has led poorly planned land use practices, encroachment of agricultural land and forests for informal settlements, increased water demand and pollution. In turn, these has caused a lot of pressure on infrastructure and natural resources such as the Sosiani river.

Sosiani River passes through Eldoret town where domestic, municipal, industrial and agricultural effluents of the town and its vicinities flow into the river [13]. The waste material from the dumpsite situated along the river is not segregated and all nature of waste is evident. There has been a drastic reduction of the river levels with little or no marine life. Car wash sheds and the Kipkenyo dumpsite along the river are the most polluters [14]. In many informal settlements, there is no regular garbage collection and no proper industrial treating devices (Kangogo [15]).

Safe drinking water accessibility brings about healthy bodies, food security, poverty reduction and extended development of a population both socially and economically [16]. People get the river water through piping to their homesteads and agricultural lands. At the homesteads the water is used for domestic consumption and livestock feeding. Consumers using water and fish caught from rivers contaminated with heavy metal face serious health consequences [17].

There is need to monitor water quality on this river on regular basis by applying and evaluating water index methods for quality assessment [18]. The objective of the present study is to assess the concentration levels of heavy metals in surface water of Sosiani River, assess the quality of the water and examine heavy metal health risk in humans. Quality indices used evaluate the water quality in Sosiani River in this study are heavy metals pollution index (HPI) and Metal Pollution index (MPI). The health risk levels from ingested water posed by heavy metals are determined using daily intake of metals and health hazard quotient and incremental life cancer risk (ILCR).

The classification of heavy metals by The International Agency for Research on Cancer [19] is based on their potential to induce carcinogenesis. The metals Cadmium (Cd), Chromium (Cr), lead (Pb), and Arsenic (As) are classified as carcinogenic, while Copper (Cu), Zinc (Zn), Iron (Fe), Nickel (Ni), Manganese (Mn), and Cobalt (Co) are classified as non-carcinogenic.

## Materials and methods

### Study area description

**Fig. 1:**
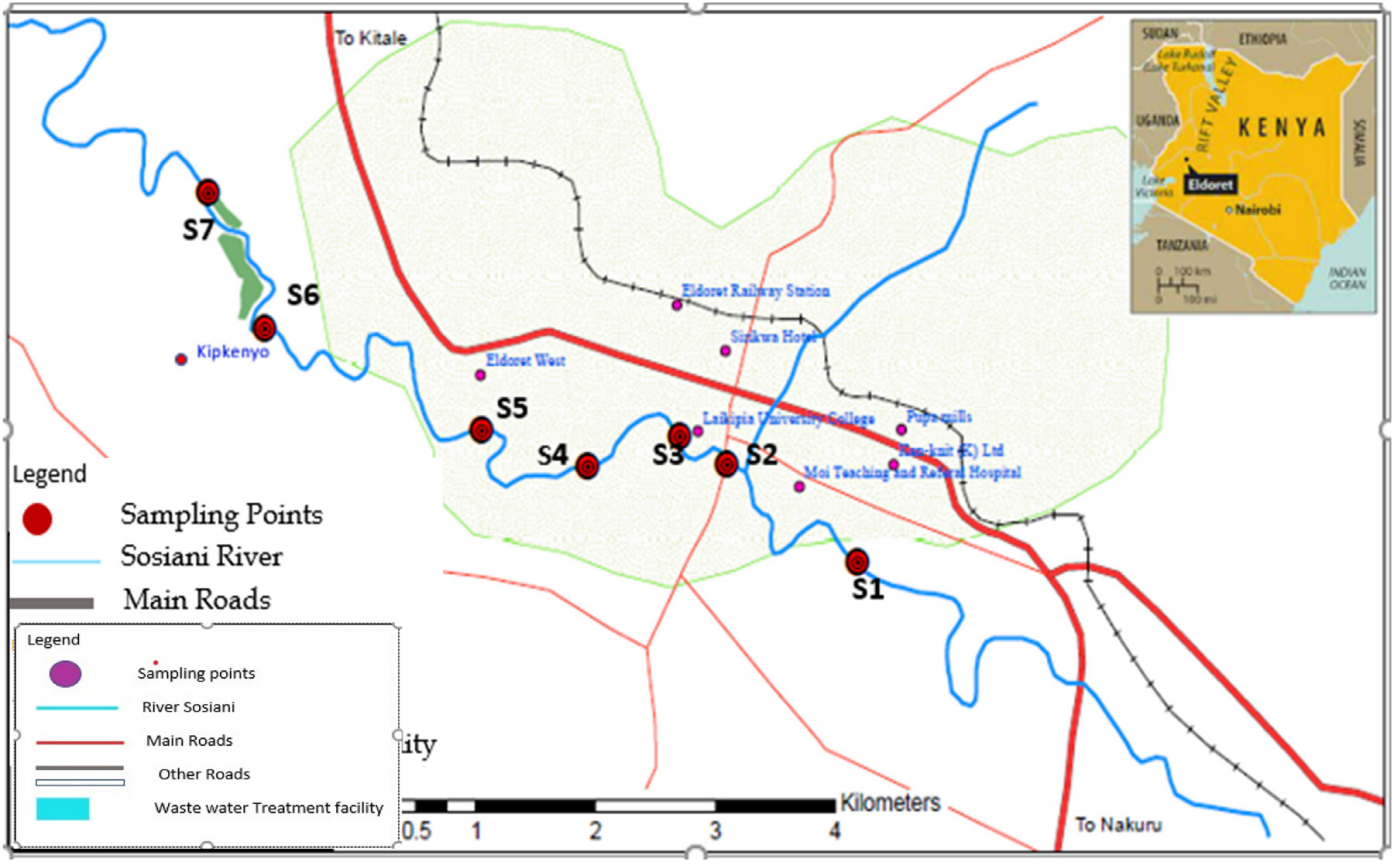
Map of the study area indicating the sampling points.

Eldoret is the capital of Uasin-Gishu County in Kenya. River passes through the town of Eldoret. Due to rise in population, urbanization has also been on the rise and this has led to the town expanding drastically and people have been forced to settle along the Sosiani River. The study area is outlined in Fig. 1.

### Sample collection and preparation

Sampling was done in the month of March at the onset of the short rains in 2018, along Sosiani River as it meandered through Eldoret town. A total of seven water samples were collected from seven stations which were described as follows: S1, MTRH (Moi Teaching Referral Hospital), which gets its water from the river; S2, Langas bridge; S3, Pioneer; S4, Kipkaren, where S3 and S4 hosts car wash, garages, scrap metal dealers, electronics and battery recyclers; S5, West Indies which is a residential suburb; S6, Kipkenyo dumpsite and S7, WWFT(Waste Water Treatment Facility).To collect the water samples, 0.5 L plastic bottles which had been pre-cleaned with distilled water and 20% HNO_3_ [20] were used. A portable GPS was used to record the longitudes and latitudes of the sampling points. Without infiltration, the collected water samples were digested with 5 ml of nitric acid to pH < 2. The acid was added to acidify and preserve the water samples.

To prepare the samples for analysis, a sample from each sampling bottle was mixed thoroughly by shaking. A 50 ml of water sample was pipetted into a digestion flask. The sample was brought to boiling slowly on a hot plate controlling the temperature at 70 °C evaporating it to about 15 ml, followed by addition of 3 ml concentrated nitric acid and 5 ml concentrated sulphuric acid while continuing heating until the solution cleared and brown fumes were no longer evident. The digested samples were cooled and filtered then topped to the mark with de-ionized water.

### Sample analysis

The seven collected samples were analyzed for three heavy metals each including lead, zinc and copper using Atomic Absorption Spectrophotometer. The mean values of the heavy metal concentration obtained in various locations were compared with the various permissible limits of the parameters set by WHO, FEPA and FAO in order to assess the quality of drinking water from Sosiani river (Table 2).

### Water quality assessment

Heavy Metal Pollution Index (MPI) evaluates the overall status of the heavy metal pollution in water and Heavy metal evaluation index (HEI) determines the overall water quality.

#### Heavy metal pollution index (MPI)

Heavy Metal Pollution Index (MPI) was determined by Eq. (1) [21].

(1) $$MPI={\left(C_{Zn}\times C_{Cu}\times C_{Pb}\right)}^{1\;/\;3}$$

where $C_{Zn},\;C_{Cu}\;and\;C_{Pb}$ are the heavy metal concentrations of metals of interest (Zn, Cu, and Pb) in mg/l.

#### Heavy metal evaluation index (HEI)

The overall water quality with regard to the heavy metal content is given by the Eq. (2) [22];

(2) $$HEI=\sum \frac{C_{i}}{MAC}$$

where MAC indicates the maximum allowable concentration and Ci is the concentration of heavy metals in the water samples.

### Health risk assessment

Risk assessment involves estimating the magnitude and nature of the harmful health impacts in humans exposed over a period of time [23]. The health risk assessment of each contaminant is based normally on the estimation of the risk level and is classified as carcinogenic or non-carcinogenic health hazards [24].

In this study, health risk was assessed by estimating the heavy metal contamination and potential carcinogenic and non-cancer health risk caused by the ingestion of heavy metals in the water from Sosiani River. Hazard Quotients (HQ), Hazard Index (HI), and the Incremental Lifetime Cancer Risk (ILCR) were used in assessing. Adults and children were considered in this case.

#### Non-cancer risks

The exposure occurs via numerous pathways like ingestion, skin absorption and inhalation. The human body ingestion is calculated from chronic daily intake (CDI). The CDI from direct ingestion of heavy metals through drinking water pathway was calculated using the Eq. (3).

(3) $$CDI=\frac{C_{i}\times IR\times EF\times ED}{BW\times AT}$$

where CDI is the chronic daily intake (mg/Kg Bw/day), Ci is the heavy metal concentration in water (mg/L), IR is the ingestion rate per unit time(1.5 L/day for adults and 0.7 L/day for children); EF is the exposure frequency (350 days/year), ED is the exposure duration (70 years and 15 years for adults and children respectively), BW is the average body weight (70 kg for adults and 15 kg for children); AT is the average exposure time which is given as ED x 365 days for carcinogens for both adults and children, AT =  30 × 365 for adults and AT =  6 × 365 for children for non-carcinogens [25].

Hazard quotient evaluates the substance potential exposure at the level where there are no adverse effects. The non-cancer risks due to non-carcinogenic effects in surface water were determined by the hazard quotient (HQ) presented in Eq. (4):

(4) $$HQ=\frac{CDI}{R_{f}D}$$

where, CDI is the chronic daily intake and R_f_D is the reference oral dose. The values of the R_f_D and cancer slope factor for different metals are listed in Table 2.

The total potential non-carcinogenic health impacts caused by exposure to a mixture of heavy metals in water was estimated using the Hazard Index (HI). This was computed according to the EPA guidelines for health risk assessment using Eq. (5) [26,27].

(5) $$HI=\sum_{k-1}^{n}HQ=HQ_{Pb}+HQ_{Zu}+HQ_{Cu}$$

The sum of all HQs of the different heavy metals in water gives an estimation of total potential health risks or the chronic health hazard. HI should not exceed one, a value ≥1 implies significant non-cancer risks, which increase with increasing value of HI [28].

#### Cancer risks

Cancer risks indicates the probability of a given population having any type of cancer as a result of ingestion of the carcinogens by determining the Incremental Lifetime Cancer Risk (ILCR).

Eq. (6) is used to compute ILCR due to exposure to a stated dose of heavy metal in drinking water [29].

(6) ILCR=CDI×CSF

CSF is the cancer slope factor which indicates the risk generated by a lifetime average amount of 1 mg/kg/day of carcinogen chemical and depends on is specific heavy metal. The CSF value for lead is 0.0085, [30]. The permissible limits are considered to be 10^−6^ and <10^−4^ for a single carcinogenic element and multi-element carcinogens [31].

## Results and discussion

### Heavy metal concentration in water

The concentration of heavy metals analysed from Sosiani River are shown in Table 1. The presence of zinc in the water can be attributed to use of zinc related fertilizers from wheat and flower farms and anthropogenic activities upstream. Chemicals used in Timber treatment and preservation factories are discharged in the river increasing the zinc metal levels in the river. Most of these factories are located along the river and neighbor the hospital.

**Table 1:** Heavy metals concentration in the current study and their comparison with selected studies in the world.

Researcher	Source	Study area	Element concentration
Present research	Surface water	Eldoret, Kenya	Cu = 0.407 ± 0.06 mg/l Zn = 0.291 ± 0.04 mg/l Pb = 0.1057 ± 0.06 mg/l
Hanaa et al. [32]	Surface water	Ismailia canal, Egypt	Pb = 2 ppm Zn = 1.90 ppm Cu = 0.2ppm
Valaei et al. [33]	Ground water	Karaj, Iran	Zn = 0.45 mg/l Cu = 2.99 mg/l Pb = 0.001 mg/l
Purushotham et al. [34]	Ground water	Maheshwaram watershed, Andhra Pradesh, India	Pd = 0.0447 mg/l Cu = 0.0433 mg/l Zn = 0.3777 mg/l
Pan et al. [35]	Ground and surface water	East China	Water (µg/l) Cu = 3.43 ± 5.44 Zn = 23.8 ± 34.9 Pb = 1.24 ± 3.61
Okey-Wokeh and Okechukwu [36]	River water	Rivers state, Nigeria	Water(mg/l) Zn = 0.015 ± 00 Cu = 0.01 ± 0.01 Pb = 0.01 ± 0.01
WHO LIMITS [37]	Drinking water Standard limit		Cu = 2 mg/l Zn = 5.0 mg/l Pd = 0.01 mg/l
FEPA [38]	Drinking water Standard limit		Cu = 1 mg/l Zn = 3 mg/l Pb = 0.01 mg/l
FAO [39]	Drinking water Standard limit		Cu = 1 mg/l Zn = 3 mg/l Pb = 0.01 mg/l
USEPA [30]	Drinking water Standard limit		Cu = 1.3 mg/l Zn = 5 mg/l Pb = 0.015 mg/l

Copper metal concentration suggests the probability of inputs of copper into the river from effluents from the textile industries, flower farms, timber treatment plants and from scrap metal operations.

The levels of lead metal are above the standard limit for drinking water. Lead levels can be brought about by point and diffuse sources from industries and urban associated activities like car washes, garages, scrap metal dealers, electronics and battery recyclers. It can also be due to pollution from town effluents and vehicle emission. There is significant spatial variation of the lead concentration which can be attributed to high degree of adsorption exhibited by lead on clay soils. Table 1 shows the elemental concentration in this study and their comparison with the selected researches in the world and their standard acceptable limit.

### Water quality indices

The Average MPI and HEI were calculated using Eqs. (1) and (2) respectively and their values shown in Table 2.

**Table 2:** Total heavy metal concentrations in water samples (mg/L), MPI and HEI.

Site	Zn	Cu	Pd	MPI	HEI
1	0.43 ± 0.06	0.32 ± 0.04	0.1 ± 0.01	0.2396	10.25
2	0.48 ± 003	0.28 ± 0.03	0.23 ± 0.02	0.3138	23.24
3	0.46 ± 0.02	0.35 ± 0.03	0.08 ± 0.02	0.2344	8.267
4	0.42 ± 0.02	0.30 ± 0.01	0.06 ± 0.01	0.1963	6.234
5	0.35 ± 0.02	0.27 ± 0.02	0.07 ± 0.01	0.1877	7.205
6	0.39 ± 0.02	0.3 ± 0.05	0.09 ± 0.03	0.2192	9.228
7	0.32 ± 0.02	0.22 ± 0.02	0.12 ± 0.01	0.1978	11.17
Mean	0.407143	0.291429	0.105714	0.2323	10.80

According to the calculated mean MPI, the water in Sosiani River can be classified as “pure water” since it lies between 0.3 and 1. The water is also classified as very highly polluted water according to the average HEI which was found to be greater than 10 [40].

### Human health risk assessment

Calculation of the Chronic Daily Intake (CDI) is the first step for non-carcinogenic analysis. As given in the Table 3, the mean values of CDI of heavy metals concentrations for adults and children were found in the order of Pb > Cu > Zn >. CDI values were higher in children compared to adults exposed to drinking water from the river.

**Table 3:** CDI, HQ and HI values of heavy metals in the river water.

Sample location	Adults				Children			
	HQ values			HI	HQ values			HI
	Zn	Cu	Pb		Zn	Cu	Pb	
S1	0.0687	0.384	1.370	1.823	0.160	0.895	3.197	4.252
S2	0.0767	0.336	3.151	3.564	0.179	0.783	7.352	8.314
S3	0.0735	0.420	1.096	1.590	0.172	0.979	2.557	3.707
S4	0.0671	0.360	0.822	1.249	0.157	0.839	1.918	2.913
S5	0.0559	0.324	0.959	1.339	0.131	0.755	2.237	3.123
S6	0.0623	0.360	1.233	1.655	0.145	0.839	2.876	3.861
S7	0.0511	0.264	1.507	1.822	0.119	0.615	3.516	4.251
Mean	0.0651	0.349	1.448	1.863	0.152	0.815	3.379	4.346
Mean CDI Values	0.01952	0.01397	0.005068		0.04555	0.03360	0.01183	

#### Non-cancer risks

Hazard quotients (HQ) and hazard indices (HI) were used to assess the non-carcinogenic risks for Zn, Cu and Pb in adults and children in the study area. The results are shown in Table 3.

The HQ and HI values for the heavy metals were also higher in children than in adults for the water source. The HQ values for Zn and Cu were within the acceptable range of less than one. The values for lead in both adults and children in the study area raises concern. A HQ value of greater1  suggests a level of concern. The HI values were greater than 1 in all sites indicating high health risk on long-term exposure and the non-cancer effect is of concern and should not be neglected. The hazard indices for children were higher compared to those of the adults implying that children could be more disposed to non-cancer risks than adults.

#### Cancer risks

The excess lifetime cancer risk of lead exposure from ingesting water from Sosiani River was estimated by Eq. (6) for both adults and children and the results presented in Table 4.

**Table 4:** ILCR values for Pb metal in Sosian River.

Sample location	Adults (10^−5^)	Children (10^−5^)
S1	1.747	3.804
S2	4.017	8.748
S3	1.398	3.043
S4	1.048	2.282
S5	1.223	2.663
S6	1.572	3.423
S7	1.921	4.184
Mean	1.846	4.021

A cancer risk value between 10^−6^ and 10^−4^ is considered to be of low health risk, and amounts greater than 10^−4^ is likely a high health risk [41]. Based on recommendations of USEPA, the carcinogenic risk range for Pb is 1.3 ×  10^−5^–3.74 ×  10^−5^ and 6.23 ×  10^−5^–1.75 ×  10^−4^ for both adults and children respectively for all the different water sources [42]. From this study, the mean ILCR values are 1.846 × 10^−5^ and 4.021 × 10^−5^ for adults and children respectively. This data indicates that there is very low cancer risk from the consumption of water from River Sosiani. There is about 19 adults and 40 children out of 1 million population each, likely to die of cancer owing to ingestion of water from Sosiani river. These results indicate higher cancer risks for children than adults.

## Conclusion

In this research the cancer and non-cancer risks of zin, copper and lead in drinking water form Sosiani River was assessed.

According to the WHO standards recommended for domestic use the mean concentration value of lead was higher. Lead metal showed high hazard indices compared with other elements in five sites signifying unacceptable non-carcinogenic risk bringing about adverse health effects. Therefore, water from Sosiani River is not safe for use domestically as far as lead metal levels are concerned and therefore the prevention and control of heavy metal contamination in the river catchment should focus on lead metal.

It is also advisable for water from the waste water treatment before being released to the river to be treated through adsorption or ion treatment to reduce the amount of lead metal in the river water.

In conclusion, there is need to monitor the effluents in the river and establish a remedy of cleaning off heavy metals from the river.

## CRediT authorship contribution statement

**Emily N. Munene:** Conceptualization, Data curation, Methodology, Writing – original draft. **Nadir O. Hashim:** Data curation, Supervision. **Willis N. Ambusso:** Writing – review & editing.

## Declaration of Competing Interest

The authors declare that they have no known competing financial interests or personal relationships that could have appeared to influence the work reported in this paper.

## Data Availability

The data that was used is shown in the downloaded manuscript in Table 3.
